# Prenatally diagnosed accessory scrotum: A case report and review of the literature on prenatal features

**DOI:** 10.1016/j.radcr.2021.12.033

**Published:** 2022-01-12

**Authors:** Koichi Deguchi, Yuko Tazuke, Miho Watanabe, Chiyoshi Toyama, Motonari Nomura, Ryuta Saka, Hiromi Harada, Yukie Nagamine, Masayuki Endo, Ritsuko Puh, Hiroomi Okuyama

**Affiliations:** a Department of Pediatric Surgery, Graduate School of Medicine, Osaka University; b Center for Maternal, Fetal and Neonatal Medicine, Osaka University Hospital; c Division of Health Sciences, Graduate School of Medicine, Osaka University; d CRIFM Clinical Research Institute of Fetal Medicine

**Keywords:** AS, Accessory scrotum, US, Ultrasonography, CT, Computed tomography, MRI, Magnetic resonance imaging, Accessory scrotum, Prenatal diagnosis, Perineal lipoma

## Abstract

Accessory scrotum (AS) is rarely diagnosed antenatally, and its prenatal features remain unknown. Here, we report a case of a prenatally diagnosed accessory scrotum with perineal lipoma. A 33-year-old woman was referred to our hospital at 35 weeks of gestation to evaluate a mass in the fetal perineal region. Prenatal ultrasonography showed a 2.0 × 2.0 cm sized, echogenic, and circular mass located posterior to the left scrotum in a male fetus. Magnetic resonance imaging (MRI) showed a mass containing adipose tissue. A 6.5 cm elastic mass (AS and protruding lipoma) was observed in the perineal region, and surgical excision was performed at 8 months of age. Histological examination confirmed the diagnosis of AS with perineal lipoma, and there was no recurrence at follow-up. The typical prenatal presentation of AS was a circular perineal mass located posterior to the normal scrotum and was associated with perineal lipoma. The prenatal detection of AS was feasible with careful observation via ultrasonography, and prenatal MRI was useful in characterizing perineal tumors and evaluating associated anomalies.

## Introduction

Accessory scrotum (AS) is a rare developmental anomaly in the perineal region, with only 53 cases reported to date [1]. Furthermore, reports on the prenatal findings of AS are few; thus, the prenatal features of AS remain largely unknown. Here, we report a case of a prenatally diagnosed accessory scrotum with perineal lipoma, which showed an atypical concentric presentation, and thus made the prenatal diagnosis difficult. We review reported prenatally diagnosed cases to summarize the prenatal findings of AS. The utility of additional prenatal imaging was also described.

## Case presentation

**Fig. 1:**
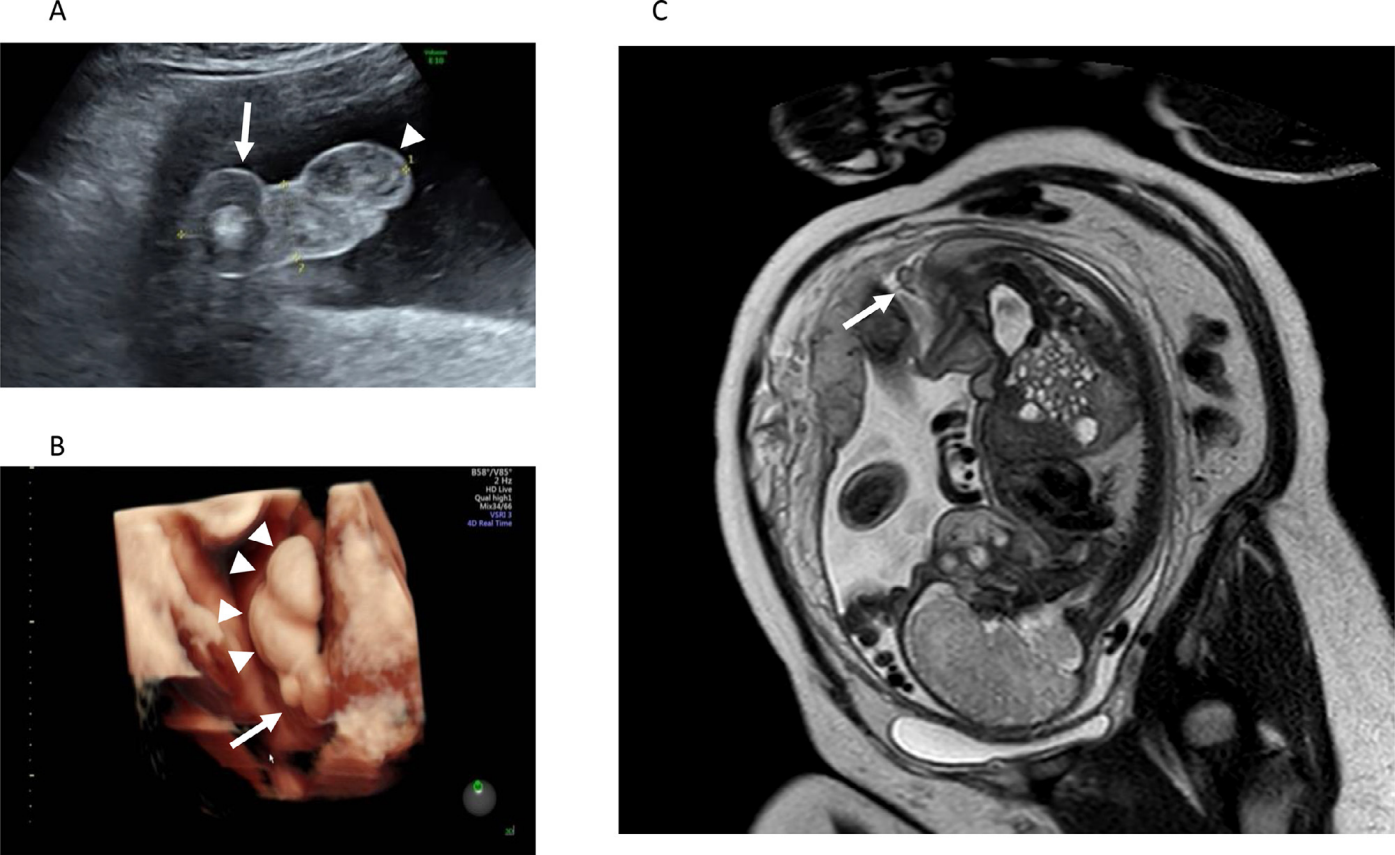
Prenatal images. (A) A prenatal ultrasonography on an axial perineal view of the perineum at 35 weeks of gestation shows a 2.0-cm-sized circular mass (arrow) posterior to the normal scrotum (arrow head). (B) The three-dimensional reconstruction image of ultrasonography shows a mass (arrow) located posterior to the normal scrotum (arrowheads). (C) A prenatal magnetic resonance image shows a perineal mass with adipose tissue characteristics (arrow).

A 33-year-old woman was referred to our hospital at 35 weeks of gestation to evaluate a mass in the fetal perineal region, which was first detected at 34 weeks of gestation at a primary care clinic. The pregnancy was uncomplicated, apart from mild iron deficiency anemia. Prenatal ultrasonography (US) revealed a 2.0 × 2.0 cm sized, echogenic, concentric circular mass located posterior to the scrotum in a male fetus (Fig. 1A–B). The echogenicity of the mass was slightly higher than that of the primary scrotum and remained unchanged during pregnancy. Magnetic resonance imaging (MRI) at 35 weeks of gestation revealed a mass with high signal intensity on T1-weighted images, and the signal intensity was suppressed on fat-suppressed T1-weighted images, suggesting the diagnosis of AS due to involvement of adipose tissue (Fig. 1C). No other abnormalities were observed. The male neonate was delivered by cesarean section at 38 weeks of gestation due to a previous cesarean delivery; his body weight was 2,952 g. No significant symptoms were noted after birth.

**Fig. 2:**
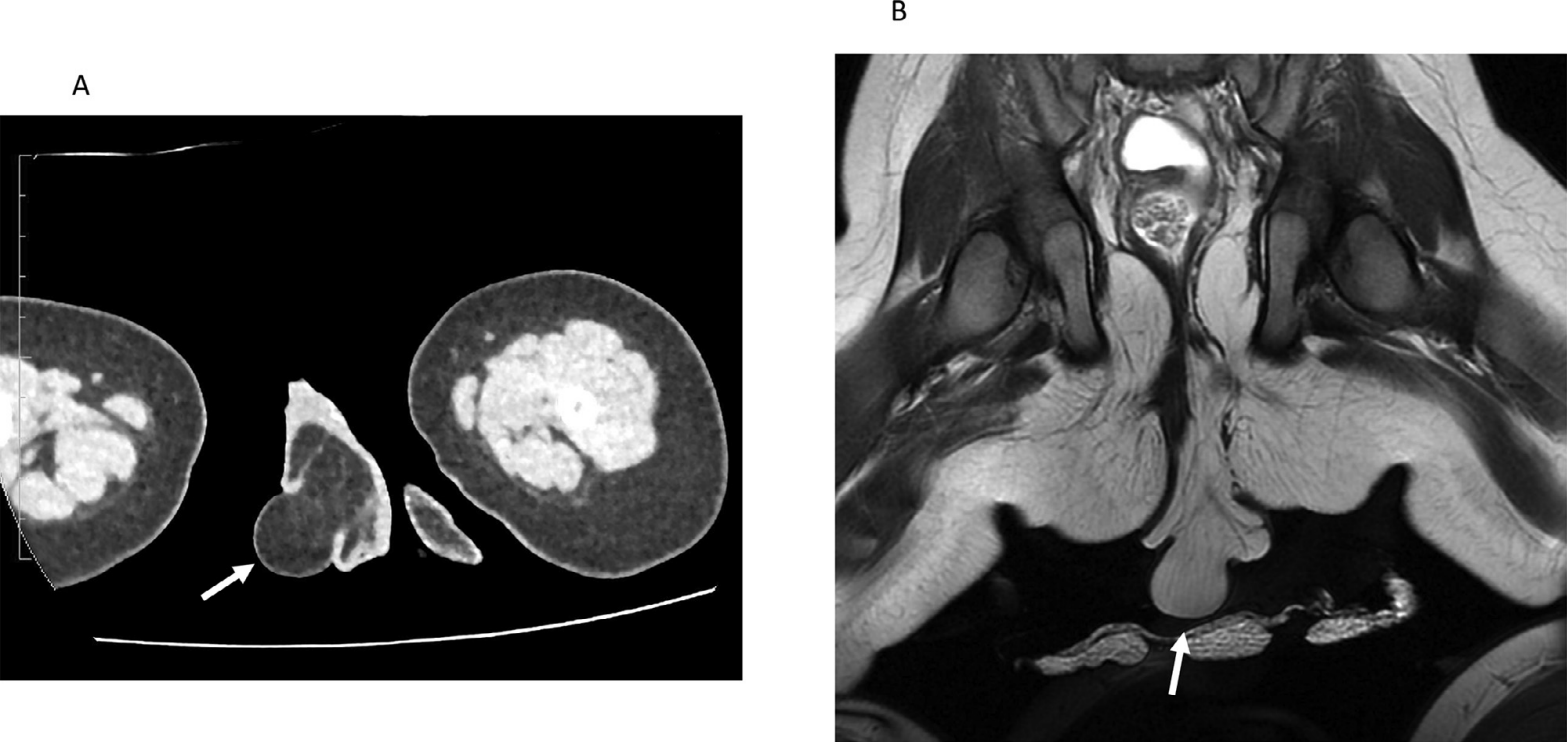
Postnatal images. (A) A computed tomography image shows an accessory scrotum with perineal lipoma. (B) A postnatal magnetic resonance image shows an accessory scrotum with perineal lipoma (arrow).

**Fig 3:**
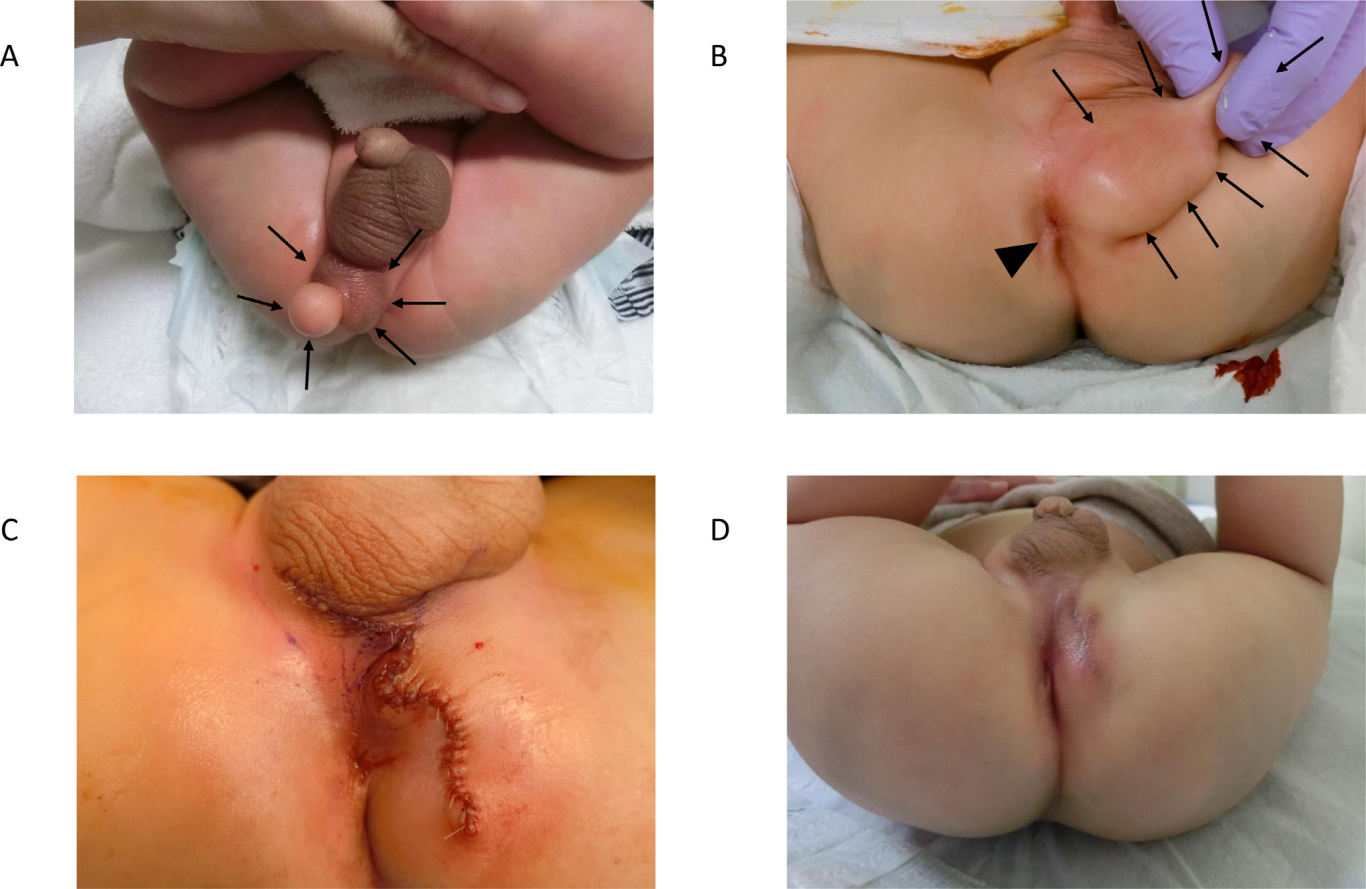
(A) Gross appearance of an accessory scrotum with perineal lipoma (arrows). (B) The association between anus (arrow head) and the accessory scrotum (arrows) was demonstrated. (C) Gross appearance after surgical resection. (D) Gross appearance of the perineal area 2 months postoperatively.

Upon physical examination after birth, his penis showed slight phallocampsis, and his scrotum appeared normal with two independent normal-sized testes. The median scrotal raphe was displaced towards the left side, as shown in Figure 3A. At the posterior-caudal area of the right scrotum, there was a 6.5 × 3.5 × 3.0 cm elastic mass, which resembled scrotum corrugations. Moreover, a 2.8 × 2.3 × 2.3 cm sized protruding lesion was located on the apex of the mass. Although his anus was located on the perineal midline and appeared normal, proximity to the mass was noted (Fig. 3B). After birth, abdominal computed tomography (CT) and MRI showed a perineal lipoma caudal to the primary scrotum and testes, and no additional associated anomalies among the intra-abdominal organs, musculoskeletal system, or genitourinary system were noted (Fig. 2).

Once normal anal function was confirmed, we resected the mass under general anesthesia at 8 months of age. The details of the procedures used are as follows: the patient was placed in the lithotomy position. A skin incision was made around the bottom of the lesion. The mass was resected along with the surrounding subcutaneous tissue without any injuries to the rectum or anus. We attempted to reconstruct the perineal raphe in the midline, which was displaced towards the right side by the mass, without excess tension (Fig. 3C). An electrical muscle stimulator was used to evaluate the location of the external sphincter muscle, which was verified to be normal. Histological examination suggested a perineal lipoma and a rugged epidermis lined with thick bundles of smooth muscle fibers. The morphology of the muscle fibers was compatible with the tunica dartos, leading to the diagnosis of an accessory scrotum with perineal lipoma.

The patient's postoperative course was uneventful, and there was no recurrence or abnormal bowel habits at 2 years of follow-up (Fig. 3D).

## Discussion

AS is a rare genital anomaly, defined as an additional scrotal-like epidermis, apart from a normally developed scrotum. AS does not contain testicular tissue and usually arises in the perineal region, typically posterior to the normal scrotum. Histologic evidence of smooth muscle fibers below the skin, compatible with tunica dartos, is diagnostic for AS [2]. We presented AS with perineal lipoma detected via third-trimester prenatal screening and performed a detailed evaluation using prenatal US and MRI. We conducted a literature review of prenatally diagnosed AS to elucidate the prenatal and postnatal courses of AS.

Although there has been a significant advancement in prenatal screening, prenatal diagnosis of AS remains rare. A literature search using PubMed identified five prenatally diagnosed AS cases, including ours [3], [4], [5], [6], and their prenatal and postnatal features are summarized. In most cases, AS was detected between the second and third trimesters: the median gestational age at detection was 31 (range: 23–35) weeks. Prenatal evaluations included US in all cases and MRI in two cases (40%). Pregnancy and delivery were uncomplicated in all cases, and their gestational ages (range: 38–39 weeks) and birth weights (range: 2208–3200 g) were within the normal range in the reported cases [7]. The median age at surgery was 3.25 months (range: 1–8), and all patients had good surgical outcomes.

Typical prenatal US features of AS in the reported cases included round, lobulated, and sometimes pedunculated masses located posterior to the normal scrotum. The echogenicity of AS was similar to that of the normal scrotum: homogenous internal echo pattern reflecting adipose tissue, with slightly higher linear echogenicity on the surface layer, which may reflect the existence of the tunica dartos [8]. In our case, the prenatal US showed an atypical concentric circular mass due to the protruding lipoma out of the AS, but other features were similar to those in previous reports. Prenatal MRI was performed in two cases (40%), including ours. Prenatal MRI of AS featured a perineal mass with adipose tissue character in both cases: a lesion with high signal intensity on T1-weighted images, which was suppressed on fat-suppressed T1-weighted images. Associated anomalies were found in all prenatally diagnosed cases (100%), including perineal lipoma in all cases, one congenital adrenal hyperplasia, one pseudodiphallia, and one phallocampsis. The incidence of associated perineal lipoma in prenatally diagnosed cases was higher than that of all AS cases, reported by Murase et al. (100% vs 72.5%) [5]. The accompanying lipoma may have aided the detection during prenatal screening. Postnatal CT and MRI were performed in two cases (40%), including ours, mainly to rule out additional anomalies, such as anorectal or genitourinary malformations. A penile nodule that was responsible for the phallocampsis was detected by the postnatal imaging in our case.

The differential diagnosis of a prenatally detected perineal mass includes lipoma, lipoblastoma, sacrococcygeal teratoma, infantile hemangioma, hamartoma, choristoma, and liposarcoma. Moreover, these lesions may be associated with anorectal and genital malformations, such as chorist Oma, labioscrotal fold, anal atresia, imperforate anus, inguinal hernia, and ambiguous genitalia [9,10]. According to a literature review by Murase et al., AS is accompanied by various anomalies, with the contiguous subcutaneous tumor being the most frequent (72.5%), followed by anorectal malformations (18.6%) and other scrotal anomalies (16.3%) [5]. Although the etiology of AS is not entirely identified, contiguous subcutaneous tumors may be related to AS development [11]. Given the wide variety of differential diagnoses and the high incidence of associated anomalies in AS, prenatal MRI allows for a better evaluation of the perineal mass and additional congenital anomalies. The association of AS with anorectal malformation is particularly important for surgical decision-making, and caution must be exercised with the sphincter muscle during surgical excision of AS. Since the AS in our case was in proximity to the anus, we assessed the patient's continence by 8 months of age according to our clinical practice in high and intermediate anorectal malformations. The electrical muscle stimulator was useful in confirming the anatomy of the anus and sphincter muscle, which was not clear via simple physical examination alone.

Herein, we report a rare case of AS with perineal lipoma. The literature review suggested that the prenatal diagnosis of AS remains challenging, but may be feasible with careful observation. Prenatal MRI was useful in characterizing perineal tumors and evaluating associated anomalies.

## Patients consent

Informed written consent was obtained from the patient for publication of the Case Report and all imaging studies. Consent form on record.

## Contributors

HH, YN, ME and RP participated in the prenatal screening and care. KD, CT, MN, RS and YT participated in the postnatal care and the operation. KD, YT and MW drafted the manuscript. OH participated in its design and coordination and helped to draft the manuscript. All authors read and approved the final manuscript.

## Authorship

All authors have made substantial contributions to all of the following: (A) the conception and design of the study, or acquisition of data, or analysis and interpretation of data, (B) drafting the article or revising it critically for important intellectual content, and (C) final approval of the version to be submitted.

## Declaration of interests

None.
